# Responses of Membranes and the Photosynthetic Apparatus to Salt Stress in Cyanobacteria

**DOI:** 10.3389/fpls.2020.00713

**Published:** 2020-06-05

**Authors:** Wenjing Yang, Fang Wang, Lu-Ning Liu, Na Sui

**Affiliations:** ^1^Shandong Provincial Key Laboratory of Plant Stress, College of Life Sciences, Shandong Normal University, Jinan, China; ^2^State Key Laboratory of Crop Biology, College of Life Sciences, Shandong Agricultural University, Tai’an, China; ^3^College of Marine Life Sciences, and Frontiers Science Center for Deep Ocean Multispheres and Earth System, Ocean University of China, Qingdao, China; ^4^Institute of Integrative Biology, University of Liverpool, Liverpool, United Kingdom

**Keywords:** cyanobacteria, membranes lipids, photosynthetic apparatus, photosynthesis, salt stress

## Abstract

Cyanobacteria are autotrophs whose photosynthetic process is similar to that of higher plants, although the photosynthetic apparatus is slightly different. They have been widely used for decades as model systems for studying the principles of photosynthesis, especially the effects of environmental stress on photosynthetic activities. Salt stress, which is the most common abiotic stress in nature, combines ionic and osmotic stresses. High cellular ion concentrations and osmotic stress can alter normal metabolic processes and photosynthesis. Additionally, salt stress increases the intracellular reactive oxygen species (ROS) contents. Excessive amounts of ROS will damage the photosynthetic apparatus, inhibit the synthesis of photosystem-related proteins, including the D1 protein, and destroy the thylakoid membrane structure, leading to inhibited photosynthesis. In this review, we mainly introduce the effects of salt stress on the cyanobacterial membranes and photosynthetic apparatus. We also describe specific salt tolerance mechanisms. A thorough characterization of the responses of membranes and photosynthetic apparatus to salt stress may be relevant for increasing agricultural productivity.

## Introduction

Salt stress is an abiotic factor that greatly influences plant survival and development. There are currently more than 1.2 billion hectares of land affected by salt, accounting for about 6% of the total global land area (Wicke et al., 2011; Yuan et al., 2016; Sui et al., 2018). Annual worldwide economic losses due to salt stress can exceed US$12 billion (Shabala, 2013; Song and Wang, 2014). Therefore, improving salt tolerance of agriculturally important plants is critical.

Cyanobacteria have a photosynthetic system similar to that of higher plants, and their cytoplasm and thylakoid membranes are similar to those of the chloroplast of higher plants in lipid composition and membrane assembly generally (Rodriguez-Ezpeleta et al., 2005; Los et al., 2010). Therefore, cyanobacteria can be used as a model system to study the mechanisms of photosynthesis, membrane lipids and signal transduction under abiotic stress (Los and Murata, 2004; Jensen and Leister, 2014), which may also provide an applicable model for higher plants. To survive in extreme or variable environment, cyanobacteria have evolved specific metabolic mechanisms and regulatory systems (Tandeau de Marsac and Houmard, 1993). For example, *Synechocystis* sp. PCC6803 (hereafter referred to as *Synechocystis* 6803), a unicellular freshwater cyanobacterium, can tolerate up to 1.2 M NaCl (Desplats et al., 2005). The photosynthetic apparatus of *Synechocystis* 6803 are similar to those of higher plants and are easier to separate (Öquist et al., 1995; Allakhverdiev and Murata, 2008). And compared with higher plants, they have relatively simple genetic systems (Haselkorn, 1991). In cyanobacteria, the thylakoid membrane is the only site of photosynthesis and also the main site of respiratory electron transport in cells (Mullineaux, 2014). The growth cycle of cyanobacteria is short, making it is easy to be treated with various salt concentrations, and there are relatively few other interfering factors. These characteristics have made cyanobacteria the ideal model system for studying the photosynthetic responses to salt stress.

**FIGURE 1:**
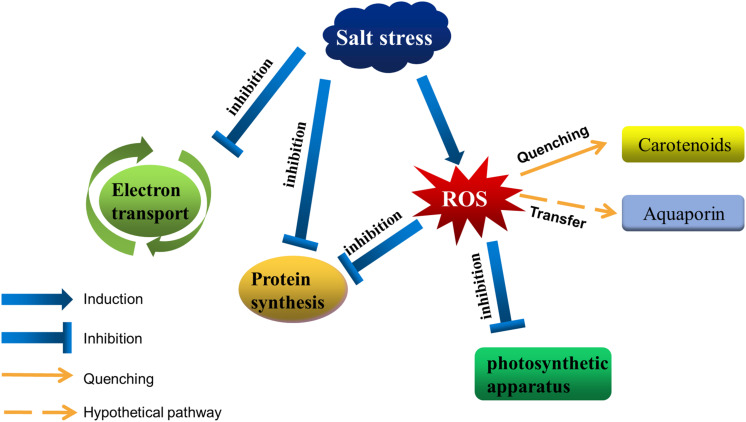
Salt stress affects photosynthetic activities in cyanobacteria. Salt stress inhibits the electron transfer around the photosystem, as well as protein synthesis. Moreover, it disrupts intracellular ion homeostasis, resulting in the accumulation of ROS in cells. Excessive ROS destroys the photosynthetic apparatus and inhibits protein synthesis. Cyanobacteria can decrease the amount of ROS by accumulating carotenoids, and aquaporins may help transfer ROS from the photosystem to other regions.

High NaCl concentrations are toxic to cells, ultimately resulting in a damaged photosynthetic apparatus (Figure 1). In cells of photosynthetic organisms, salt stress leads to a decrease in cell volume and osmotic stress, it also inhibits the photosynthetic electron transfer process (Allakhverdiev and Murata, 2008; Feng et al., 2014; Zhao et al., 2019). 0.5 M NaCl inactivated both PSII and PSI in *Synechococcus* cells due to the changes in K/Na ratio (Allakhverdiev et al., 2000). In spirulina platensis, salt stress leads to a reduction in PSII electron transport by increasing the number of Q_B_-non-reduction reaction centers (Lu and Vonshak, 1999). After that, it was be found that salt stress did not directly affect the PSII activity itself in the dark, but the same salt stress combined with photosynthesis effective radiation blocked electron transport between Q_A_ and Q_B_ (primary and secondary quinone electron acceptors of PSII located in proteins D2 and D1, respectively), and the degree of inhibition was proportional to the intensity of light (Lu and Zhang, 2000; Lu and Vonshak, 2002). Sudhir et al. (2005) found that NaCl not only inhibited PSII activity by inhibiting the D1 protein, but also decreased energy transfer from light harvesting antenna to PSII by changing other thylakoid membrane proteins such as 47 kDa chlorophyll protein (CP) and 94 kDa protein in *Arthrospira platensis*. Intracellular sodium and potassium homeostasis is important for maintaining the normal activity of enzymes (Hu et al., 2016; Chen et al., 2017; Ma et al., 2019), cell membrane potential (Zhang et al., 2014; Wei et al., 2017), and normal cell volume (Serrano and Rodrigueznavarro, 2001; Zhu, 2003; Shao et al., 2014). The low concentration of Na^+^ helps to regulate the pH in plants and cyanobacteria, as well as the fixation of nitrogen and carbon dioxide (Karandashova and Elanskaya, 2005; Han et al., 2012; Fan et al., 2019). Excessive Na^+^ and Cl^–^ flows into the cell disrupted ion homeostasis, leading to accumulation of reactive oxygen species (ROS) (Scarpeci et al., 2008; Feng et al., 2015; Song et al., 2020). High ROS levels are toxic to cells, with the associated destruction of the photosynthetic apparatus and membrane lipid peroxidation (Zhang et al., 2012; Sui and Han, 2014; Yang et al., 2020), adversely affecting photosynthesis. Additionally, high NaCl concentrations inhibit the *de novo* synthesis of proteins, including many photosystem-related proteins, such as the D1 protein (Allakhverdiev et al., 2002). For example, ROS produced in cells under salt stress could inhibit transcription factor activities, resulting in downregulated *psbA* expression (Jimbo et al., 2018).

Cyanobacteria have evolved diverse physiological mechanisms to cope with salt stress. Cyanobacteria may alter the content of certain membrane proteins in order to repair or resynthesize photosynthetic complexes damaged by salt stress. For instance, salt stress results in the enhancement of the vesicle-inducing protein in plastids 1 (*Vipp1*) in *Synechocystis* 6803 (Huang et al., 2006). The *Vipp1* have been suggested to be involved in the assembly of the thylakoid membrane system by transferring lipids from the inner envelope of chloroplast (Li et al., 1994; Vothknecht et al., 2012). It has also been found that photosystem I (PSI) and photosystem II (PSII) reaction center cores are originally synthesized in the plasma membrane of cyanobacteria (Zak et al., 2001). Therefore, the increased *Vipp1* may help accelerate the transport of the reaction center cores to thylakoid membrane under salt stress (Huang et al., 2006). Marin et al. reported that the acclimation of *Synechocystis* 6803–684 mM NaCl (4%, w/v) can be divided into two phases: the initial response to salt shock and longer-term adaptation to salt (Marin et al., 2004). In the first phase, the expression levels of many genes are upregulated, whereas in the second stage, the expression levels of only a few of these genes remain upregulated, such as the genes involved in glucosylglycerol (GG) synthesis and those encoding ABC transporters (Marin et al., 2004). Kanesaki et al. revealed that the expression of many genes is induced in response to salt stress, including the genes associated with the D1 protein at the photochemical reaction center of PSII (Kanesaki et al., 2002; Qiao et al., 2013). For instance, the *rbp3* gene, which encodes a type II RNA-binding protein, helps maintain the transcript levels of acyl-lipid desaturase genes (*desA*, *desB*, and *desD*) and the substantial abundance of unsaturated membrane lipid (Tang et al., 2010). This review focuses on the effects of salt stress on membrane, the photosystem and photosynthetic activities of cyanobacteria. We also describe the mechanisms in cyanobacteria that facilitate the protection of photosystems from the detrimental effects of salinity stress.

## Effects of ROS on Photosynthesis Under Salt Stress

The O_2_ molecule has two unpaired electrons that have the same spin quantum number (Gill and Tuteja, 2010). Due to its spin restriction, molecular oxygen prefers to receive electrons from high energy level to produce the so called ROS which are toxic to cells at high concentration (Ahanger et al., 2017). ROS includes singlet oxygen (^1^O_2_), superoxide anion (O_2_^–^), hydrogen peroxide (H_2_O_2_), and the hydroxyl radical (OH⋅) etc. In plant, peroxisomes not only destroy ROS due to the activities of peroxidases and catalases, but also produce superoxide radicals and there are, at least, two sites of superoxide generation: one in the organelle matrix, the generating system being xanthine oxidase, and another site in the peroxisomal membranes dependent on NAD(P)H (del RÍo et al., 2002). Thus in plants, chloroplasts and peroxisomes are the main producers of ROS during the day, while mitochondria are the main producers at night (Moller, 2001; Foyer and Noctor, 2003; Gill and Tuteja, 2010). In cyanobacteria, ROS are also mainly derived from photochemical reactions and the photosynthetic electron transport (Latifi et al., 2009; Wada et al., 2013). In the process of photosynthetic electron transfer, ROS are mainly produced at three sites (Asada, 1999): (1) The oxygen-evolving complex in the water photolysis releases ROS due to state III inactivation. (2) The electrons are lost to O_2_ by PQ on the reducing side of PSII to produce H_2_O_2_. (3) The electrons are transferred to O_2_ directly or by ferredoxin (Fd) to produce O_2_^–.^ on the reduction side of PSI. Under high light stress, due to the limited acceptors in the respiratory chain, electron leakage leads to the reduction of triplet oxygen (^3^O_2_) into O_2_^–.^ (Mittler, 2002). Studies have shown that cyanobacteria cells under salt stress have a huge demand for ATP synthesis, which may reduce the fixation rate of CO_2_ and lead to the excessive reduction of ferroredoxin pool (Van Thor et al., 2000; Latifi et al., 2009). This may be one of the reasons why salt stress result in oxidative stress.

Under abiotic stress conditions, singlet chlorophyll is transformed into triplet chlorophyll, which transfers its energy to ^3^O_2_ to form ^1^O_2_. ^1^O_2_ has a short half-life in the cell and reacts with nearby target molecules (proteins, pigments, and lipids) immediately (Gorman and Rodgers, 1992; Latifi et al., 2009). It has been shown that ^1^O_2_ and H_2_O_2_ can inhibit the translational elongation of *psbA* mRNA (Nishiyama et al., 2001, 2004). And H_2_O_2_ also interrupts the energy transfer between the core and the terminal emitter of phycobilisomes in *Synechocystis* 6803 (Liu et al., 2005). Excessive ROS will lead to membrane lipid peroxidation, which not only directly affects the normal function of cells, but also aggravates oxidative stress by producing lipid-derived free radicals (Montillet et al., 2005). ROS reacts with large molecules such as phospholipids and enzymes in the membrane to form lipid peroxidation products, resulting in decreased membrane fluidity (Gill and Tuteja, 2010). And ROS damages membrane proteins and ion channels, causing leakiness of some substance.

The scavenging of ROS in cyanobacteria cells is mainly by energy dissipation, antioxidant enzymes and non-enzymatic antioxidants. For instance, energy dissipation can be carried out by the “CP43′ protein”, and this process is induced by iron deficiency in cyanobacteria (Latifi et al., 2009). Cyanobacteria can also dissipate energy through the Mehler-like reaction. Mehler first described the process of reducing O_2_ to H_2_O_2_ by photosynthetic electron transport chain in chloroplast, and this reaction is therefore called the Mehler reaction (Mehler, 1951; Mehler and Brown, 1952). After that, H_2_O_2_ is rapidly detoxified to water by the ascorbate peroxidase pathway. In this process, the electrons are split from the water in oxygen releasing complex (OEC) and then flow through the PSI to produce water again. Therefore, it is called the water-water cycle, or “pseudocyclic electron flow” (Asada, 1999, 2000). Compared with plant, there is a similar reaction in cyanobacteria, known as the Mehler-like reaction. This reaction can reduce O_2_ with electrons mediated by PSI by means of soluble flavoproteins 1 and flavoproteins 3 proteins (Vicente et al., 2002; Helman et al., 2003; Helman et al., 2005; Allahverdiyeva et al., 2011; Allahverdiyeva et al., 2013). It is a four-electron transfer reaction and it does not produce ROS and may protect PSI against production of O_2_^–^ (Allahverdiyeva et al., 2013). The superoxide dismutase (SOD), most effective intracellular enzymatic antioxidant, can remove O_2_^–^ by catalyzing its dismutation: one O_2_^–^ being reduced to H_2_O_2_ and another oxidized to O_2_ (Gill and Tuteja, 2010). Kanesaki et al. demonstrated that salt stress and hyperosmotic stress induced the expression of *sodB*, which encoded an FeSOD enzyme (Kanesaki et al., 2002). Non-enzymatic antioxidant carotenoids can protect cellular structures, especially the photosystems, from the damage by scavenging ^1^O_2_ and excitation energy. Carotenoids are a group of highly reductive substances that can protect cellular structures, especially the photosystems, from the damage by scavenging ^1^O_2_ and excitation energy. In photosynthetic organisms, carotene mainly has three functions: harvesting light energy, acting as a light screen, and quenching singlet oxygen (Frank and Cogdell, 1996). In thylakoids, the carotenoids have two functions: harvesting light energy and photoprotection via the quenching of energy and ^1^O_2_ (Sedoud et al., 2014). The orange carotenoid protein (OCP) is a soluble blue-green photoactive protein that binds a single keto-carotenoid molecule (Kirilovsky and Kerfeld, 2013, 2016; Dominguez-Martin and Kerfeld, 2019). And salt stress increases its transcripts and proteins levels (Kanesaki et al., 2002; Fulda et al., 2006). In cyanobacteria, OCP interacting with the phycobilisome, increases energy dissipation in the form of heat, thereby decreasing the amount of energy arriving at the reaction centers (Wilson et al., 2006). Kawasaki et al. (2013) found a purified astaxanthin-binding OCP from a eukaryotic microalga, named AstaP, shows high solubility in water and can quench singlet oxygen. And they found that the gene encoding AstaP was significantly up-regulated by salt stress (Kawasaki et al., 2013). A lack of β-carotene prevents cyanobacteria from generating a functional PSII, whereas a lack of almost all xanthophylls resulted in a considerable increase in the intracellular ROS content (Sozer et al., 2010; Zhu et al., 2010). Thus, in response to environmental stress, including salt stress, carotenoids are critical for maintaining the stability of photosynthesis. In addition to the above, aquaporins may also be involved in the scavenging of ROS under salt stress. Some aquaporin isoforms mediate permeation of glycerol, H_2_O_2_ or CO_2_ in addition to water (Maurel, 2007). Some plant aquaporins expressed in yeast can transport H_2_O_2_ molecules (Bienert et al., 2007), implying that aquaporins may be able to maintain the stability of photosystems by transferring ROS to other regions. The Δ*aqpZ* cells of *Synechocystis* 6803 showed defects in macronutrient metabolism, pH homeostasis, and cell division under photomixotrophic conditions (Akai et al., 2011), but whether the above mechanism applies to cyanobacteria remains further experimental exploration.

In some cyanobacteria such as PCC6803 which contain polyunsaturated fatty acids, ROS would react with the acyl chains of polyunsaturated fatty acids (PUFA) or their membrane lipid residues, triggering the chain reaction of lipid peroxidation (Fryer, 1992; Yang et al., 2008). In order to maintain the normal physiological function of cells under salt stress, cyanobacteria not only need to remove excessive ROS, but also the oxidation products of ROS, such as lipid peroxides. It has been showed that tocopherol and carotenoids were very important for the scavenging of lipid peroxides in *Synechocystis* 6803 (Maeda et al., 2005). The content of tocopherol in cyanobacteria increased under high light and decreased when glucose was added to the medium (Backasch et al., 2005). This effect also showed that tocopherol takes part in protection against oxidative damage.

## Effects of Salt Stress on Polypeptide Composition of Photosystem

Salt stress could inhibit protein synthesis, with the D1 protein among the most obviously affected. In the natural environments, salt stress often occurs concomitantly with high light stress. Many studies have focused on the effects of salt stress on cyanobacterial PSII under intense light (Allakhverdiev and Murata, 2004; Allakhverdiev et al., 2005; Jantaro et al., 2005; Murata et al., 2007). A previous study revealed that exposure to 0.5 M NaCl can inhibit the repair of photodamaged PSII in *Synechocystis* 6803, but it did not directly accelerate the photodamage to PSII (Murata et al., 2007). It was also indicated that 0.5 M NaCl suppressed the synthesis of the D1 protein at the translational level (Murata et al., 2007). Allakhverdiev and Murata demonstrated that the initial rate of photoinactivation is unaffected by various NaCl concentrations, but the initial recovery rate decreases by 50 and 100% in the presence of 0.5 M and 1.0 M NaCl, respectively (Allakhverdiev and Murata, 2004). By monitoring the incorporation of [^35^S]Met into the thylakoid membrane proteins under the recovery conditions, they observed that the synthesis of the D1 protein and many other proteins was completely impeded by 1.0 M NaCl (Allakhverdiev and Murata, 2004). Jantaro et al. (2005) determined that under severe osmotic stress conditions, the abundance of the PSII D1 protein in *Synechocystis* 6803 was decreased, whereas the protein contents of PsaA/B and NdhF3 were unaffected. They also proved that osmotic stress is more detrimental to photosynthesis than ionic stress. The D1 protein appeared to be one of the proteins targeted by osmotic stress conditions (Jantaro et al., 2005).

**FIGURE 2:**
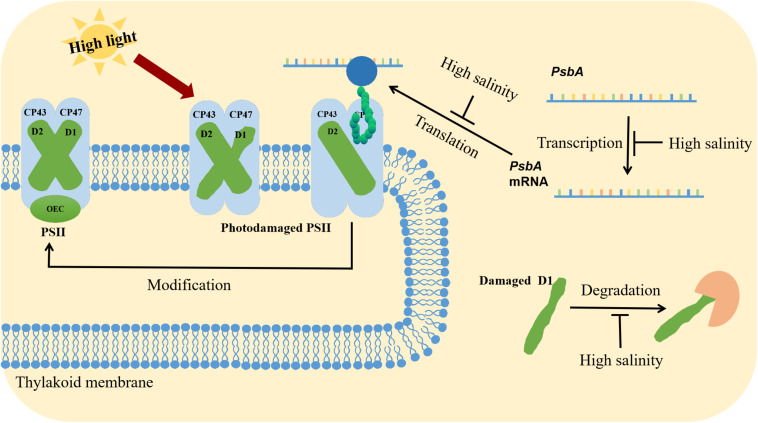
Synergistic effects of high light and salinity stress on PSII. High light stress destroys the PSII structure. The repair of PSII involves the degradation of the D1 protein, the transcription, and translation of *psbA*, the incorporation of pre-D1 into PSII, the processing of pre-D1, and the assembly of the PSII dimer and the oxygen-evolving complex. Salt stress inhibits *psbA* transcription and translation.

The repair of PSII involves the degradation of D1, transcription of *psbA* genes, translation of *psbA* transcripts, incorporation of pre-D1 into PSII, processing of pre-D1, as well as the assembly of the PSII dimer and the oxygen-evolving complex (Figure 2; Murata et al., 2007). Ohnishi and Murata reported that salt stress inhibited not only protein synthesis but also the degradation of the D1 protein in the photodamaged PSII of *Synechococcus elongatus* PCC 7942 (hereafter *Synechococcus* 7942), with betaine alleviating both of these inhibitory effects (Ohnishi and Murata, 2006; Nishiyama and Murata, 2014). Allakhverdiev et al. (2002) observed that the translation of mRNAs might be the primary cellular process inhibited by a decrease in the intracellular ATP level. Transcription was only partially affected by the ATP content and was likely to be a secondary target (Jantaro et al., 2005). During translation, the extension of polypeptide chains and the transfer of amino acids require energy from ATP, making ATP synthesis a potential rate-limiting step for the complete repair of photodamaged PSII (Jantaro et al., 2005).

Nishiyama et al. (2001, 2004) reported that ROS could inhibit the elongation step of the translation of *psbA* mRNA, which encodes the D1 protein in *Synechocystis* 6803. Biochemical studies reveal that ROS destroyed EF-G and EF-Tu, which regulates translational elongation (Kojima et al., 2007; Yutthanasirikul et al., 2016; Jimbo et al., 2018). Specifically, EF-G translocated peptidyl-tRNA from the A site to the P site of the ribosome, and it forms an intramolecular disulfide bond when inactivated by H_2_O_2_ (Kojima et al., 2009). In contrast, EF-Tu delivered aminoacyl-tRNA to the A site of the ribosome, and when inactivated by H_2_O_2_, it formed sulfenic acid and an intermolecular disulfide bond (Yutthanasirikul et al., 2016). Intense light conditions could enhance *de novo* protein synthesis of a *Synechocystis* 6803 mutant, whose Cys-82 targeted by ROS is replaced by a Ser residue (Jimbo et al., 2018). Thus, it is reasonable that excessive ROS contents induced by environmental stress can inhibit translation by disrupting transcription factor structures.

Many experiments have confirmed that salt stress inhibits *de novo* protein synthesis during the PSII repair process in cyanobacteria, but in addition to the above studies, the effects of salt stress on the synthesis of other proteins in photosynthetic apparatus remain to be studied. And besides ROS stress, the other effects of salt stress (such as ion stress and osmotic stress) on protein synthesis also need to be further studied. Three hypotheses have been proposed (Murata et al., 2007). First, the influx of NaCl into the cell may directly inhibit protein synthesis by destabilizing polysomes and ribosomes (Tester and Davenport, 2003; Murata et al., 2007). It was previously indicated that Rubisco of *Tamarix jordanis* was inactivated in response to high NaCl concentrations (Solomon et al., 1994). Thus, another hypothesis is that the primary target of salt stress is Rubisco. Salt stress inhibits the fixation of CO_2_ and diminishes the regeneration of acceptors for the linear electron transport thereby inducing the generation of ROS, which subsequently inhibits protein synthesis (Murata et al., 2007; Nishiyama and Murata, 2014). The third hypothesis is that an increase in the intracellular NaCl concentration inactivates ATP synthesis, thereby decreasing the intracellular ATP content that is essential for protein synthesis (Allakhverdiev et al., 2005). In addition to these hypotheses, we speculate that salt stress may also indirectly inhibit protein synthesis by inducing ROS production.

## Effect of Lipid Composition and Fatty Acid Desaturation on the Salt Resistance of Cyanobacteria

The cell membrane is a sensor of environmental stress, which can activate a series of protective reactions by adjusting the stress perception and rigidity of cell structure (Sui et al., 2018). In cyanobacteria and plants, the thylakoid membrane structure and fluidity were affected by lipid composition and the degree of fatty acid desaturation (Mikami and Murata, 2003). The relationship between lipids and salt stress has been widely studied in plants and cyanobacteria. Sui et al. demonstrated that salt stress increases the content of unsaturated fatty acids in the euhalophyte *Suaeda salsa*, which increased the photosystem tolerance to salt stress (Sui et al., 2017). In the halophyte *Thellungiella*, increasing the content of phosphatidylglycerol (PG) and sulfoquinovosyl diacylglycerol (SQDG) as well as the ratio of monogalactosyldiacylglycerols/digalactosyldiacylglycerol (MGDG/DGDG) in thylakoid membranes can alleviate PSII photoinhibition under salt stress (Sui and Han, 2014).

In response to salt stress, cyanobacteria could alter not only the composition of lipids but also the unsaturated degree of membrane lipids. The changes in lipid composition of *Synechococcus* sp. PCC6311 under salt stress include the increase of the proportion of unsaturated fatty acids and phosphatidyl glycerol (Huflejt et al., 1990). Ritter and Yopp (1993) observed that the proportion of mono/digalactosyl diacyl glycerols changes when exposed the cyanobacterium *Aphanothece halophytica* to salt stress. Changes in ion-exchange properties of the cytoplasmic membrane caused by changes in polar lipids may hamper the function of potassium ion channels (Joset et al., 1996), thereby reducing the inflow of Na^+^ and reducing the damage of photosynthetic apparatus. The decrease in the protein to lipid ratio led to increased cytochrome oxidase and H^+^/Na^+^ antiport activities in *Synechococcus* 6301 under salt stress (Peschek et al., 1994; Srivastava et al., 2013), which also reduced the damage of photosynthetic apparatus.

The extent of the desaturation of individual fatty acids is regulated genetically and environmentally, and the content of unsaturated fatty acids will influence the photosynthetic machinery under salt stress. *Synechocystis* 6803 contains four acyl lipid desaturases, DesA, DesB, DesC, and DesD, which catalyze the desaturation at the Δ12, Δ15, Δ9, and Δ6 positions of C18 fatty acyl chains in membrane lipids, respectively (Murata and Wada, 1995; Chintalapati et al., 2006). The oxygen-evolving machinery in thylakoid membranes isolated from the *desA^–^/desD^–^* cells of *Synechocystis* 6803 (the double mutant of *desA* and *desD* genes) was more sensitive to NaCl than that of the WT cells, indicating that the desaturation of membrane lipid fatty acids may directly protect the oxygen-evolving machinery against salt-induced inactivation (Allakhverdiev et al., 1999). In order to further study on the function of *desA*, Allakhverdiev et al. (2001) constructed *desA*^+^ cells by overexpressing the *desA* gene in *Synechococcus* 7942 that do not have the *desA* gene. The results showed that WT cells are more sensitive to NaCl and have the reduced capacity of recovery than *desA*^+^ cells (Allakhverdiev et al., 2001). These studies showed that the increase of unsaturated fatty acids in membrane lipids enhances the tolerance of the photosynthetic and Na^+^/H^+^ anti-port systems in cyanobacteria to salt stress (Allakhverdiev et al., 2001; Singh et al., 2002).

The desaturation of fatty acids in membrane lipids may protect the oxygen-evolving machinery by stimulating the synthesis of the protein(s) in the Na^+^/H^+^ antiport system (Allakhverdiev et al., 1999). The energy generated by photosynthesis and respiration overcomes the inhibition of protein synthesis by NaCl, thus enabling the repair of the oxygen-releasing complex. The following scheme might explain why light or glucose can restore the oxygen-evolving activity (Allakhverdiev et al., 1999). The lack of an ATP supply under the dark and salt conditions indirectly inhibited the activities of the Na^+^/H^+^ antiport system and caused sodium ions to flow into the thylakoid cavity, leading to the deactivation of the oxygen-releasing complex. The presence of NaCl in the dark impeded the synthesis of certain proteins, whereas exposure to light has the opposite effect. The above-mentioned results implied that the affected proteins belong to the Na^+^/H^+^ antiport system. Compared with the WT Na^+^/H^+^ antiport system, the mutant was more sensitive to salt and its repair rate was slower (Allakhverdiev et al., 1999). Therefore, unsaturation of fatty acids might also stimulate the synthesis of protein(s) in the Na^+^/H^+^ antiport system.

The *rbp3* gene, which encodes a type II RNA-binding protein, is involved in maintaining the transcript levels of acyl-lipid desaturase genes (*desA*, *desB*, and *desD*) and the substantial abundance of unsaturated membrane lipids (Tang et al., 2010). It was shown that 2-day treatment with 1 M NaCl decreased the photosynthetic activity of Δ*rbp3* to 8.4% of the WT level (Wang and Xu, 2013). The complementation with *rbp3* fully restored the photosynthetic activity, whereas the overexpression of *desA* only partially restored the activity (Wang and Xu, 2013). Thus, *rbp3* may maintain the extent of unsaturated lipids in the cell membrane by enhancing the stability of *des* mRNAs, thereby stabilizing the photosynthesis of cyanobacteria under salt stress conditions.

## Conclusion and Perspectives

Salt stress inhibits protein synthesis, especially the D1 protein. Osmotic stress may directly inhibit protein synthesis. Moreover, it seems that the accumulated ROS caused by salt stress mainly inhibits the translation process. However, whether salt stress affects the modification processing of pre-D1 in cyanobacteria remains unclear.

Aquaporins may be able to stabilize photosystems by transferring ROS to other regions, since some plant aquaporins expressed in yeast can transport H_2_O_2_ molecules (Bienert et al., 2007). However, the function of aquaporins and the associated mechanisms in cyanobacteria remits further characterization.

Cyanobacteria represent an excellent model system for studying the relationship between membrane systems and photosynthesis under stress conditions. Several studies have revealed the protective effects of membrane lipids and unsaturated degree of fatty acids on photosystems under salt stress, but the protective effects of the composition of different lipids on the photosynthetic apparatus of cyanobacteria under salt stress remains to be further studied. Whether salt stress affects the lipid-assisted PSI oligomerization and affects energy transfer between phycobilisomes and the photosystem also requires further exploration. Although there is increasing interest in uncovering the effects of salt stress on photosynthetic activities in cyanobacteria, the underlying mechanism remains to be elucidated.

## Author Contributions

WY initiated preparation of the manuscript. NS, FW, and L-NL conceptualized the idea and revised the manuscript. All authors have read and approved the final manuscript.

## Conflict of Interest

The authors declare that the research was conducted in the absence of any commercial or financial relationships that could be construed as a potential conflict of interest.
